# Associations between sleep and circadian disruption in shift work and perpetration of interpersonal violence

**DOI:** 10.3389/frsle.2023.1220056

**Published:** 2023-10-19

**Authors:** Rosalie B. Flinn, Rebecca M. C. Spencer

**Affiliations:** ^1^Department of Psychological and Brain Sciences, University of Massachusetts, Amherst, MA, United States; ^2^Institute for Applied Life Sciences, University of Massachusetts, Amherst, MA, United States

**Keywords:** stress, alcohol, circadian rhythm, aggression, anger

## Abstract

Research has uncovered substantial consequences of shift work on health outcomes through disruption of sleep and circadian rhythms. Less explored is how the effects of shift work on sleep and circadian rhythms can facilitate interpersonal aggression and violence within the home (i.e., intimate partner violence, child abuse). Given challenges in direct studies on this topic, integration across related literature is critical. In this narrative review, we identify compounding variables centered around sleep and circadian rhythms that place shift workers at an increased risk of perpetrating interpersonal violence. Shift workers have impaired sleep and altered circadian rhythms. Associated alternations in executive functioning, stress, and alcohol use provide pathways to increase risk for interpersonal violence. A model of interactions facilitating the relationship between shift work and interpersonal violence is proposed to promote prevention efforts and motivate policy change.

## 1. Introduction

Interpersonal violence (IPV) is the intentional use of physical force or power against others via physical, psychological, sexual, or neglectful means (Mercy et al., 2017). Experiencing violence within the household has detrimental long-lasting impacts. Between 17% and 39% of the population experience physical IPV each year (Capaldi et al., 2012) and IPV is a leading cause of death among young adults (Plummer et al., 2017). Child maltreatment occurs at a rate of 8.1 out of every 1,000 children, most frequently in the form of neglect or physical abuse (Mulder et al., 2018). Given the high prevalence of IPV, understanding risk factors for perpetrating IPV is essential.

One particular risk for IPV may be shift work. Shift work refers to the rotation of workers to ensure 24-h coverage (Costa, 2016), resulting in many employees with irregular work arrangements (permanent night shifts, consistent unstructured work hours; Åkerstedt, 1990). Over 25% of the working population in the United States and 15–20% in Europe is involved in shift work with about 7% engaged in regular night work (Straif et al., 2007; Centers for Disease Control and Prevention, (CDC) and National Institute for Occupational Safety and Health, (NIOSH), 2015; Costa, 2016).

The nature of shift work exposes these individuals to poor sleep, high stress (Marquié et al., 2015), and strain on personal relationships in marriages and families (Presser, 2000; Davis et al., 2008; Skoufi et al., 2017), all of which may put one at risk for committing IPV. Existing studies have reported on the relationship between sleep and violence (Paiva and Canas-Simião, 2022); the association between poor sleep and high subjective stress and aggression (Demichelis et al., 2022); occupational violence victimization and sleep problems (Magnavita et al., 2019); hostility as a predictor of sleep quality and quantity (Granö et al., 2008); the correlation between poor sleep and aggression or violence (Kamphuis et al., 2012, 2014); intensified anger following sleep restriction (Krizan and Hisler, 2019); and how sleep problems contribute to aggression (Krizan and Herlache, 2016). Missing from this research are the effects of misaligned circadian rhythms and socioeconomic status on violence and anger. This review expands on existing literature, providing a novel perspective. Specifically, the purpose of this review is to distill evidence that shift workers are at an increased risk of perpetration of IPV given compounding factors related to sleep and circadian disruption. Given the difficulty of studies directly on perpetration of violence, it is essential to draw from this literature on related topics, as reviewed here. A summary of reviewed literature appears in Table 1.

**Table 1:** Summary of research articles in review.

**Reference**	***n***	**Theme**	**Sub-theme(s)**	**Results**
Acheson et al. (2007)	20	Sleep deprivation	Impulsive behavior	One night of sleep deprivation caused a significant increase in impulsive responses among women, but not among men
Bani-Issa et al. (2020)	335	Shift work	Stress, cortisol, sleep quality	Night shift work and a shift lasting longer than 8 h were significantly associated with impaired morning and bedtime levels of cortisol among female healthcare professionals
Barker et al. (2016)	95, 141	Sleep	Aggression	Male prisoners reported greater reactive and proactive aggression if they perceived themselves to have poor sleep
Brum et al. (2022)	36	Shift work	Cortisol, melatonin, circadian rhythm	Night shift workers demonstrated reduced levels of salivary cortisol during their shifts and on off-days
Cannizzaro et al. (2020)	90	Shift work	Cortisol, stress	Cortisol levels were significantly altered among a sample of security guards on the night shift, showing increases before and after their shift
Chang (2018)	132	Shift work	Sleep quality, cortisol, stress	Type of shift influenced sleep quality and cortisol levels, with evening and night shift nurses experiencing the greatest impacts
Charles et al. (2016)	319	Shift work	Cortisol, stress	Police officers working primarily night shifts showed a significantly shallower slope of salivary cortisol
Davis et al. (2008)	1,166	Shift work	Stress, family satisfaction	Night shift work was associated with greater reported levels of marital instability and work spillover into family time
Eyy et al. (2019)	166	Sleep	Risk-taking	Higher sleep need perception predicted more risk taking among habitual sleepers of short duration
Ganesan et al. (2019)	52	Shift work	Sleep, melatonin, alertness	A lack of circadian adaptation to night work led to impaired alertness and performance. Working during peak urinary melatonin secretion was associated with greater subjective sleepiness and impairment
Garefelt et al. (2020)	3,706	Sleep, stress	Insomnia symptoms	Perceived and occupational stressors predicted reported issues in initiation and maintenance of sleep, while trouble maintaining sleep was associated with increased levels of perceived and occupational stress
Grandner et al. (2013)	4,081	Sleep	Sociodemographic traits, socioeconomic status	Self-reported sleep symptoms were associated with different respective sociodemographic and socioeconomic characteristics
Granö et al. (2008)	5,433	Sleep	Hostility	Greater hostility was associated with increased sleep disturbances and may contribute to shorter sleep duration
Hafner et al. (2017)	62,000	Sleep	Economic cost	Data from OECD nations indicated substantial economic losses due to inadequate sleep. Poor sleep is both a public health and economic issue.
Han et al. (2021)	38	Shift work	Intervention, naps	Nurses allowed a scheduled nap during a 12-hour shift reported less fatigue and provided a higher level of clinical care
Haynes et al. (2006)	23	Sleep, aggression	Substance use, intervention	Aggressive ideation and self-reported aggressive acts were reduced following a behavioral sleep intervention among adolescents using substances
Hoshino et al. (2009)	83	Sleep	Domestic violence, aggression	Women experiencing domestic violence reported more sleep symptoms. Half of respondents reported greater aggressiveness in their partner following a poor night's sleep
Kadiani et al. (2020)	50	Domestic violence	Profile of perpetrators	Domestic violence perpetrators reported suffering from an average of 4 adverse childhood experiences, being alcohol-dependent, and symptoms of anxiety/depression
Kamphuis et al. (2014)	96	Sleep	Aggression, impulsivity	Poorer sleep quality and more insomnia symptoms were associated with higher self-reported impulsivity and aggression in forensic psychiatric patients
Kim et al. (2019)	827	Shift work	Psychological wellbeing	Shift workers reported greater stress, more insomnia symptoms, poorer quality of life, and more anxiety compared to non-shift workers
Koshy et al. (2019)	11	Shift work	Circadian rhythms	Police officers working the night shift demonstrated disrupted peripheral circadian rhythms
Krizan and Hisler (2019)	142	Sleep	Anger, affect	Sleep restriction over 2 days increased feelings of anger and resulted in negative affect
Lammers-van der Holst et al. (2021)	29	Shift work, circadian rhythm	Intervention	A bright-light exposure treatment mediated circadian disruption in a laboratory-simulated night shift work routine
Li et al. (2018)	70	Shift work	Cortisol	Shift work altered diurnal cortisol secretion among junior physicians, predicting subsequent increased cortisol release
Marquié et al. (2015)	3,232	Shift work	Cognition, long-term impact	Chronic shift work incurred a dose-dependent negative impact on cognition capacity
Pakyurek et al. (2002)	2	Sleep disorders	Aggression	Two inpatient children demonstrated significant reduced aggression following treatment of their moderate sleep and breathing disorders
Plescia et al. (2021)	392	Shift work	Alcohol, insomnia	Night-shift workers were more likely to consume alcohol, particularly binge-drinking, compared to day-shift workers
Presser (2000)	3,467	Shift work	Family stability	The impact of shift work on family relations was dependent upon the presence of children in the household.
Rajaratnam et al. (2011)	4,957	Sleep, sleep disorders	Shift work, police officers	Police officers assigned to the night shift frequently met criteria for a sleep disorder
Rauer and El-Sheikh (2012)	430	Intimate partner violence	Sleep	Psychological intimate partner violence was strongly associated with sleep quality in both men and women. Sleep problems predicted future perpetration of intimate partner violence
Razavi et al. (2019)	130	Shift work	Melatonin	Chronotypes and schedule of rotating shift influenced levels of melatonin
Shin et al. (2005)	4,695	Trait anger	Sleep	Sleep disturbance and disruptions were associated with high levels of trait anger
Skoufi et al. (2017)	91	Shift work	Quality of life, work-life conflict	Nurses reported higher rates of burnout and conflict between family and work life, regardless of shift work status, compared to non-nurses
Sladek et al. (2020)	61	Stress, rumination	Sleep	Higher daily stress levels were correlated with shorter sleep duration and higher cortisol levels upon waking
Smith et al. (2021)	15	Sleep	Cognition	Chronic sleep restriction over 6-weeks suggested restriction led to accumulation of sleep debt and a small to moderate negative impact on cognition
Swanson et al. (2016)	22	Night-shift work, circadian misalignment	Alcohol-related health problems	Night shift workers may be at an increased risk of health issues from alcohol use because of central circadian misalignment
Turchi et al. (2019)	197	Night-shift work	Quality of life	Nurses working night-shift reported significantly poorer health-related quality of life than general public
Vaughn et al. (2015)	2,499	Sleep	Aggression	Poor sleep was associated with reactive violence among African-Americans
Watanabe et al. (2022)	49	Night-shift work	Interventions to reduce sleepiness symptoms	Night shift workers who took a nap of ≥ 90 min exhibited lower drowsiness following nap breaks and less fatigue at the end of night shift compared to those with shorter naps.
Wilson et al. (2017)	86	Short sleep duration	Inflammation, interpersonal responses	When both partners experienced short sleep duration, couples were more hostile to each other
Wright et al. (2015)	17	Sleep deprivation, circadian misalignment	Cortisol levels	Total sleep deprivation and chronic circadian misalignment appeared to influence cortisol levels
Yong et al. (2017)	6,338	Shift work	Sleep problems	Short sleep duration, poor sleep quality, and insomnia were commonly reported by night shift workers
Zhang and Lei (2023)	455	Aggression	Sleep quality	A causal relationship between subjective sleep quality and aggressive behavior was supported
Zhang et al. (2020)	435	Shift work	Cortisol, sleep disorders, neural mechanisms	Shift work is associated with the higher levels of cortisol and a greater risk of sleep disorders

## 2. Shift work on sleep and circadian rhythms

Sleep is theorized to be regulated by two independent yet complementary processes: an endogenous circadian clock and a homeostatic sleep mechanism (Borbély et al., 2016). Timing of sleep is highly moderated by circadian rhythms, akin to a pacemaker, while homeostatic mechanisms control sleep depth and maintenance (Deboer, 2018). Sleep is optimized when an individual sleeps in accordance with their circadian rhythm and with an appropriately high level of sleep pressure.

Sleep deprivation and disruption are the most recognized health outcomes of shift work. Boivin and Boudreau (2014) reported that shift employees experience reduced sleep time, reduced sleep quality and symptoms of insomnia. Poor sleep is greatest in night shift workers (Yong et al., 2017). To compensate for an inability to sleep during typical sleeping hours overnight, many shift workers adopt a biphasic sleeping schedule, supplementing an inadequate sleep period with a nap >1 h. However, complete compensation for lost sleep is difficult in situations of chronic sleep restriction (Boivin et al., 2022).

The shift in the circadian timing of sleep can change sleep quality as well. Sleeping outside one's optimal circadian schedule can further reduce sleep quantity and change the architecture (physiological stages) of sleep. Night shift workers have daytime sleep duration 1–4 h shorter than other shift workers' nighttime sleep (Pilcher and Coplen, 2000). Early morning shift workers also experience reduction in sleep duration and excessive sleepiness upon waking (Åkerstedt and Wright, 2009). Related changes in sleep architecture include increased stage 1 non-REM sleep while stage 2 and REM sleep are reduced (Åkerstedt, 1998).

Shift workers commonly turn to alcohol before bed under the belief it will induce sleep (Shriane et al., 2020) when, instead, alcohol may yield further sleep disruptions. Approximately 90% of shift workers consume alcohol regularly (Kivimaki et al., 2010) and 17% of night shift workers use alcohol specifically to relieve insomnia (Brown et al., 2020). In comparison to day workers, rotating and night shift workers are more likely to utilize alcohol as a sleep aid (Richter et al., 2021). Counter to the colloquial belief, alcohol disrupts sleep architecture and promotes insomnia (He et al., 2019). While alcohol can reduce sleep onset time, sleep is heavily disrupted particularly in the second half of night (Thakkar et al., 2015). Alcohol delays REM sleep onset and reduces REM sleep time in a dose-dependent manner. Tolerance can develop within three nights, thus requiring higher doses to incur both sedative (reduced sleep onset) and sleep stage alteration effects (Roehrs and Roth, 2001). As such, alcohol use begets dependence, which begets sleep disruption, motivating further alcohol ingestion. Alcohol withdrawal also disrupts sleep, making this cycle difficult to stop (Thakkar et al., 2015).

Socioeconomic contexts may further exacerbate sleep problems in shift working populations. Many shift-work jobs are low-paying and do not require advanced education (with some notable exceptions, e.g., doctors and pilots). Most night shift workers earn <$31,500 per year (U.S. Bureau of Labor Statistics, 2019). Sleep inequalities are prevalent in low socioeconomic status (SES) populations (Grandner et al., 2013, 2016; Jehan et al., 2018), including shift workers. Low SES is associated with shorter sleep time, high sleep fragmentation and wake after sleep onset (Etindele Sosso et al., 2021). Low SES neighborhoods predispose residents to poor sleep via physical environmental factors (i.e., ambient noise, artificial light) and social environmental factors (i.e., neighborhood violence, lack of social cohesion) which negatively impact sleep (Kim et al., 2022).

Often neglected are the negative outcomes of shift work due to circadian misalignment. Circadian misalignment refers to the desynchronization between circadian clocks and the environment (Boivin et al., 2022). Non-standard work hours and shift work require swift alterations in sleep-wake routines, and sleep-wake rhythms out of sync with the light-dark cycle. Stable circadian rhythms allow the body to optimize efficiency of behaviors by aligning physiological functions with behavioral demands (Arendt, 2010). Thus, circadian desynchrony has numerous detrimental consequences including sleep disruptions (described above), metabolic disorders, poor mental health, cognitive impairments, and cancer risk.

Night-time light and a lack of daytime light exposure are the primary causes of circadian misalignment in night shift workers (Smolensky et al., 2015). Night work necessitates exposure to nocturnal artificial light, directly disrupting circadian rhythms regulating sleep. Melatonin release is inhibited by light and triggered by darkness (Arendt, 2010) which is critical to the stabilization of circadian rhythms (Pévet, 2002). Night shift workers were found to have a 15% reduction in salivary melatonin levels compared to day workers (Daugaard et al., 2017; Wei et al., 2020). Likewise, night-shift nurses have smaller nadir melatonin levels, delayed nadir onset, and lower melatonin overall (Razavi et al., 2019). The peak of melatonin release will typically occur during work for night shift employees. This results in sleepiness on the job and the reduction of sleep quality during the daytime sleep period (Ganesan et al., 2019; Boivin et al., 2022).

Cortisol release is also regulated by the circadian clock and is dysregulated in shift workers. Cortisol secretion typically peaks around waking and reaches nadir early in the night. Lower cortisol levels are conducive to sleep but occur during working hours of many shift workers (Boivin and Boudreau, 2014; Boivin et al., 2022). Night shift work may reduce the magnitude of cortisol secretion (Koshy et al., 2019; Boivin et al., 2022). However, other studies find increased cortisol from shift work (Li et al., 2018; Cannizzaro et al., 2020; Zhang et al., 2020). Cortisol aids in the entrainment of peripheral circadian clocks and may regulate behavioral adaptation to shift in circadian phase (Lo Martire et al., 2020).

Finally, sleeping out of phase with the circadian rhythm also alters sleep architecture. Stage two non-REM sleep and REM sleep are reduced in all shift workers (Åkerstedt, 1998; Åkerstedt and Wright, 2009). Cortisol may contribute to this, as infusions of cortisol are shown to decrease REM sleep (Kim et al., 2015; Lo Martire et al., 2020). Importantly, reduced REM sleep duration is associated with impaired mood and emotional dysregulation (Cartwright, 2005; Naiman, 2017).

Overall, there are clear sleep and circadian disruptions from shift work, particularly night shift work. The nature of shift work also provides exacerbating factors such as alcohol use and nighttime light exposure which contribute to physiological changes in sleep and hormone secretion. We next turn to the behavioral implications of disrupted sleep and circadian rhythms, particularly violence-related behaviors.

## 3. Effects of disrupted sleep and circadian rhythms on violence-related behaviors

Sleep and circadian desynchronization have been connected to anger, aggressive behavior and violence. Shorter sleep duration and lower sleep quality are associated with higher anger and aggression (Krizan and Hisler, 2019; Van Veen et al., 2021, 2022). Unethical behavior is also positively related to poor sleep (Barnes et al., 2011). For example, police officers with a chronic sleep disorder were more likely to show maladaptive work-related behavior (e.g., uncontrolled anger toward others; Rajaratnam et al., 2011). Sleep problems or disturbance have been directly associated with interpersonal conflict and aggression. Rauer and El-Sheikh (2012) examined the reciprocal pathways between interpersonal conflict (e.g., psychological aggression) and sleep, finding significant correlations between sleep problems in men and women and perpetration of psychological interpersonal conflict.

Conversely, when aggressive and violent behaviors are observed, they are often associated with poor sleep. For example, adults engaged in fist-fights were more prone to high dissatisfaction with their sleep (Vaughn et al., 2015). Likewise, men who commit domestic violence are more likely to be sleep deprived and demonstrate increased aggression after a night of poor sleep (Hoshino et al., 2009). Moreover, forensic psychiatric patients with chronic insomnia are more hostile and aggressive (Kamphuis et al., 2014).

Anger, aggression, and IPV may be more likely in shift workers due to heightened stress (Kim et al., 2019; Brown et al., 2020). Many shift workers face a confluence of psychosocial stressors and may have an impaired ability to handle these stressors (Arlinghaus et al., 2019; Fischer et al., 2019). Shift work is associated with reduced quality of life, high work-life conflict, and a negative impact on social and family life (Skoufi et al., 2017; Turchi et al., 2019). Shift work also causes social jet lag, the discrepancy in sleep time typically for weekdays vs. weekends, but, more broadly, for workdays vs. non-workdays. This stress manifests itself as poor sleep and highly variable sleep times in this population (Arlinghaus et al., 2019; Fischer et al., 2019).

Sleep deprivation and restriction are themselves stressful, which underlies a reciprocal relation between sleep and stress (Medic et al., 2017; Garefelt et al., 2020). Acute episodes of sleep restriction and stress both increase cortisol including in shift workers (Demichelis et al., 2022; Grosser et al., 2022). Chronic stress generally has the opposite effect, decreasing the cortisol response to stress (Thau et al., 2022). As such, long-term shift workers may suffer from chronic stress and thus reduced levels of cortisol. For example, studies in female healthcare professionals found 36% of night-shift workers had reduced morning cortisol levels (Bani-Issa et al., 2020). Night and evening shift workers had a lower cortisol awakening response compared to day shift workers (Chang, 2018). Likewise, police officers frequently working the night shift displayed impaired cortisol slopes (Charles et al., 2016). Importantly, low cortisol is associated with aggressive behavior and some research has found a negative correlation between cortisol and aggression (Bronsard and Bartolomei, 2013).

Alcohol intake to “treat” impaired sleep and relieve stress in shift workers (Richter et al., 2021) presents another risk for violent behavior. Several studies note a higher frequency of binge or heavy drinking behavior in nurses and industrial shift workers. Night shift workers, who use alcohol more frequently than day shift workers (Plescia et al., 2021), may experience greater impacts from alcohol use (Swanson et al., 2016). Alcohol can promote interpersonal animosity and raise the potential for violence (Sontate et al., 2021). Assaults tend to be more violent when alcohol is involved. Based on samples of IPV perpetrators, a majority regularly consume alcohol or are alcohol dependent (Hoshino et al., 2009; Kadiani et al., 2020). There is an increased risk of IPV occurrences when either or both partner(s) uses any psychoactive substance (Trezza and Popp, 2000). Thus, increased alcohol use due to the nature of shift work can increase levels of aggression by reducing inhibitory control and heightening emotional reaction.

Executive functioning is also directly impaired in chronic sleep restriction such as that experienced by shift workers (Lowe et al., 2017). Sleep loss is negatively correlated with behavioral inhibition (Satterfield and Killgore, 2019) and impulsivity (Anderson and Platten, 2011). Persistent short sleep is associated with risky decision-making (Eyy et al., 2019). Mechanistically, sleep restriction has a detrimental impact on prefrontal cortex functioning (Owens and Weiss, 2017; Smith et al., 2021), which shows diminished metabolic activity after sleep deprivation (Satterfield and Killgore, 2019). Negativity bias is stronger following sleep loss which increases impulsive reactions to negative stimuli (Acheson et al., 2007; Anderson and Platten, 2011). The heightened arousal elicited by perceived threats is compounded by the lack of inhibitory control induced by insufficient sleep.

Regulation of emotions also becomes more difficult with restricted sleep. Emotional instability manifests as a short temper, greater irritability and anger (Shin et al., 2005; Granö et al., 2008; Kamphuis et al., 2012; Krizan and Hisler, 2019). Disrupted sleep enhances feelings of depression, anxiety and suicidality (Satterfield and Killgore, 2019). Insomniacs consistently reply with greater anger to hypothetical provocative situations compared to healthy sleepers (Kamphuis et al., 2012). Greater levels of aggression and hostility are predicted by a shorter sleep duration (Van Veen et al., 2022), restricted sleep (Shin et al., 2005), reduced sleep quality and insomnia (Kamphuis et al., 2014). This lack of emotion regulation, plus a reduced ability to recognize others' emotions, increases inflammatory responses to interpersonal conflicts (Wilson et al., 2017; Satterfield and Killgore, 2019). Given these factors, persistent sleep deficits in shift workers put them at risk for emotional outbursts and violent behavior toward those around them.

Finally, risk of violence may be increased with more time awake at night. Coming and going from a night shift and being more awake at night on days off may put night shift workers at greater risk of committing violence for several reasons. First, circadian influences on emotionality. For instance Tucker et al. (2012) found that emotional reactivity was greatest at off-peak (relative to their chronotype) times of day. Other studies point to circadian and chrontype-related changes in violent behavior as further evidence that perpetration of violence may be association with circadian disruption (Hood and Amir, 2018).

## 4. Discussion

Interpersonal violence is a global issue affecting millions of individuals and families, the prevalence of which is likely much higher than any recorded statistics due to lack of reporting. The harm caused by IPV necessitates investigation into all possible angles of prevention and intervention. Beyond economic benefits (Costa, 2016; Hafner et al., 2017), policies that reduce sleep and circadian disruption in shift workers may prevent or reduce the risk for perpetrating IPV.

Together, the reviewed research points to the risk of perpetration of IPV in shift workers. Shift workers experience sleep and circadian disruption and are also susceptible to the compounding factors of sleep loss and stress which amplify anger, aggression and violence. Disrupted circadian rhythms and chronic sleep restriction raise stress levels, increase alcohol use, and impair executive functioning. Low sleep efficiency, shorter total sleep time, and circadian dysruption are associated with violence-related behaviors (Van Veen et al., 2021, 2022), which may manifest in interpersonal relationships and potentially cause violence (Kamphuis et al., 2012).

**Figure 1:**
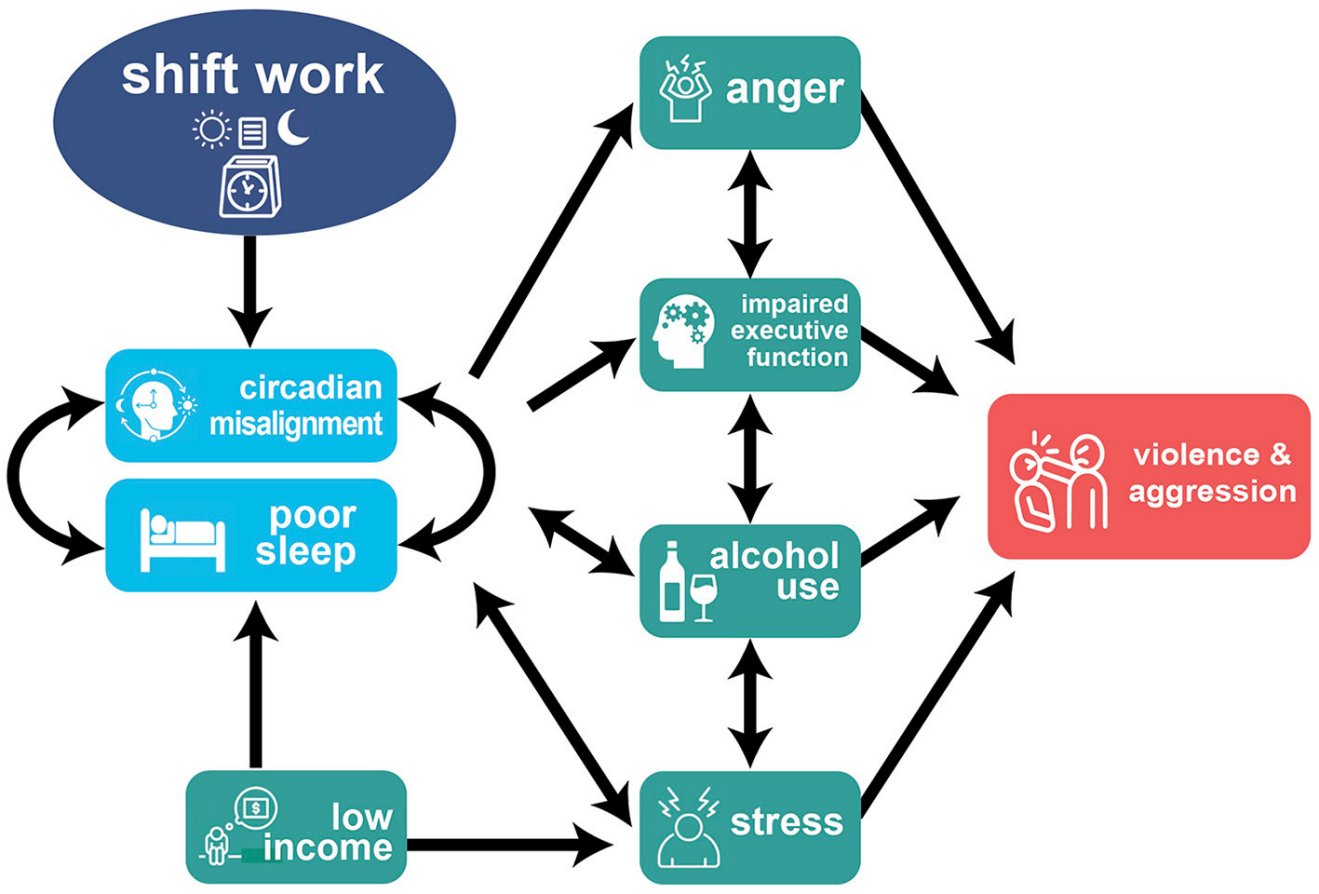
Proposed model of the pathway from shiftwork to interpersonal violence.

Krizan and Herlache (2016) proposed a three-path model linking sleep loss to aggression. By this account, sleep loss: (1) increases negativity bias, (2) increases perceived hostility, and (3) lowers inhibitory abilities. We provide an expanded model specific to shift workers (Figure 1), illustrating the complex factors in the relationship between shift work and violence and aggression. This cycle exacerbates stress (Kim et al., 2019; Brown et al., 2020) and alcohol use (Richter et al., 2021) while impairing executive function (Marquié et al., 2015) and generating angry feelings (Krizan and Herlache, 2016; Krizan and Hisler, 2019).

Interventions aimed at improving sleep and circadian rhythms have been shown to reduce anger and violent outbursts. For instance, children with breathing-related sleep disorders demonstrated reduction in aggressive and violent behavior following treatment (Pakyurek et al., 2002). Likewise, improvement in sleep time in adolescents treated for substance use was likewise associated with lower aggressive ideation and action (Haynes et al., 2006). Thus, improving sleep and circadian health in shift workers is likely to be effective in reducing risk for IPV.

Common approaches to sleep and circadian health of shift workers have focused on optimizing shift schedules. Permanent night shift and rotating 12-h shift workers show the greatest impairment in sleep quality, sleep duration and the most social jet lag (Casjens et al., 2022) with a clockwise rotational schedule providing more favorable outcomes (Burgess, 2007). Other approaches include light exposure treatment (Lammers-van der Holst et al., 2021) and allowing naps on shifts (Han et al., 2021; Patterson et al., 2021; Watanabe et al., 2022). Other potential interventions include the use of exogeneous melatonin and education on sleep habits (Åkerstedt and Wright, 2009). Targeting other aspects of risk, including alcohol use, also shows promise for reducing violence (Laslett et al., 2022).

Importantly, not all shift workers will commit acts of anger and violence. However, given the severity and potential for these outcomes and the difficulty in studying them, the proposed model provides multiple avenues toward intervening on workers at great risk of violent outcomes. Moreover, although such research is difficult, this review points for a greater need for data on shifts of employment (not just location of employment) for perpetrators.

## Author contributions

RF developed the initial concept and literature review and writing of the paper. RS contributed to the further conceptual development and writing. All authors contributed to the article and approved the submitted version.
